# Rapid Replacement of SARS-CoV-2 Variants by Delta and Subsequent Arrival of Omicron, Uganda, 2021

**DOI:** 10.3201/eid2805.220121

**Published:** 2022-05

**Authors:** Nicholas Bbosa, Deogratius Ssemwanga, Hamidah Namagembe, Ronald Kiiza, Jocelyn Kiconco, John Kayiwa, Tom Lutalo, Julius Lutwama, Alfred Ssekagiri, Isaac Ssewanyana, Susan Nabadda, Henry Kyobe-Bbosa, Jennifer Giandhari, Sureshnee Pillay, Upasana Ramphal, Yajna Ramphal, Yeshnee Naidoo, Derek Tshiabuila, Houriiyah Tegally, Emmanuel J. San, Eduan Wilkinson, Tulio de Oliveira, Pontiano Kaleebu

**Affiliations:** Medical Research Council/Uganda Virus Research Institute, Entebbe, Uganda (N. Bbosa, D. Ssemwanga, H. Namagembe, J. Kiconco, J. Kayiwa, T. Lutalo, J. Lutwama, A. Ssekagiri, P. Kaleebu);; London School of Hygiene and Tropical Medicine Uganda Virus Research Unit, Entebbe (D. Ssemwanga, R. Kiiza, J. Kiconco, J. Kayiwa, T. Lutalo, J. Lutwama, A. Ssekagirl, P. Kaleebu);; Ministry of Health, Kampala, Uganda (I. Ssewanyana, S. Nabadda, H. Kyobe-Bbosa);; University of KwaZulu-Natal, Durban, South Africa (J. Giandhari, S. Pillay, U. Ramphal, Y. Ramphal, Y. Naidoo, D. Tshiabuila, H. Tegally, E.J. San, E. Wilkinson, T. de Oliveira);; Stellenbosch University, Stellenbosch, South Africa (E. Wilkinson, T. de Oliveira)

**Keywords:** coronavirus disease, COVID-19, severe acute respiratory coronavirus 2, SARS-CoV-2, coronaviruses, viruses, variants, Delta, Omicron, respiratory infections, rapid replacement, genomic surveillance, whole-genome deep sequencing, zoonoses, Uganda

## Abstract

Genomic surveillance in Uganda showed rapid replacement of severe acute respiratory syndrome coronavirus 2 over time by variants, dominated by Delta. However, detection of the more transmissible Omicron variant among travelers and increasing community transmission highlight the need for near–real-time genomic surveillance and adherence to infection control measures to prevent future pandemic waves.

Severe acute respiratory syndrome coronavirus 2 (SARS-CoV-2) is the etiologic agent of human coronavirus disease (COVID-19), which was declared by the World Health Organization to be a global pandemic in March 2020 (*1*). Since the beginning of the pandemic, COVID-19 has caused enormous socioeconomic destruction (*2*) and has resulted in >5 million deaths worldwide.

A study conducted by the Medical Research Council/Uganda Virus Research Institute (MRC/UVRI) and the London School of Hygiene and Tropical Medicine (LSHTM) Uganda Research Unit (Entebbe, Uganda) during the early phase of the pandemic showed that most SARS-CoV-2 infections were imported and consisted of several lineages that included A, B, B.1, B.1.1, B.1.1.1, and B.4 (*3*). A subsequent study that covered the period from December 2020 through January 2021 showed that a SARS-CoV-2 lineage A variant (A.23.1) had emerged and become the dominant variant in Uganda (*4*).

The UVRI and its partners, such as the MRC/UVRI and LSHTM, contribute to the SARS-CoV-2 response in Uganda. As part of routine national genomic surveillance, we identified circulating variants during June‒December 2021 and analyzed trends of SARS-CoV-2 lineages over time.

## The Study

We conducted SARS-CoV-2 whole-genome deep sequencing for 266 nasooropharyngeal samples collected during June‒December 2021 from 28 travelers arriving at Entebbe International Airport and from 238 patients in Uganda from 18 districts (Kampala, Wakiso, Mpigi, Kalungu, Kalangala, Dokolo, Amudat, Moroto, Kassanda, Gulu, Arua, Koboko, Amuru, Lamwo, Kwania, Apac, Kisoro, and Mityana). All samples had tested positive for SARS-CoV-2 by reverse transcription PCR with cycle threshold values <30 and were sequenced by using Illumina MiSeq (https://www.illumina.com) (n = 236, 88.7%) and Oxford Nanopore MinION (https://nanoporetech.com) (n = 30, 11.3%) next-generation sequencing platforms. Most (77%) samples sequenced were from the central region of Uganda (mostly from Kampala, Wakiso, Mpigi, and Kalungu); fewer samples came from the northern (13.9%) and western regions (8.2%) of the country.

We assembled deep sequence reads by using the genome detective software (*5*) (for the Illumina MiSeq‒generated sequence reads) and Nanopolish/Medaka (https://artic.network/ncov-2019/ncov2019-bioinformatics-sop.html) (for the Nanopore‒generated sequence reads) to obtain high-quality SARS-CoV-2 genomes with >80% coverage. We performed quality control of all sequences to check for adequate coverage, indels, and frameshifts. We performed mutation calling by using Nextclade (https://clades.nextstrain.org), followed by SARS-CoV-2 lineage analysis with Pangolin (https://github.com/cov-lineages/pangolin). To analyze trends of SARS-CoV-2 lineages over time, we downloaded all sequences from Uganda in GISAID (https://www.gisaid.org) (950 sequences as of January 10, 2022).

**Figure 1:**
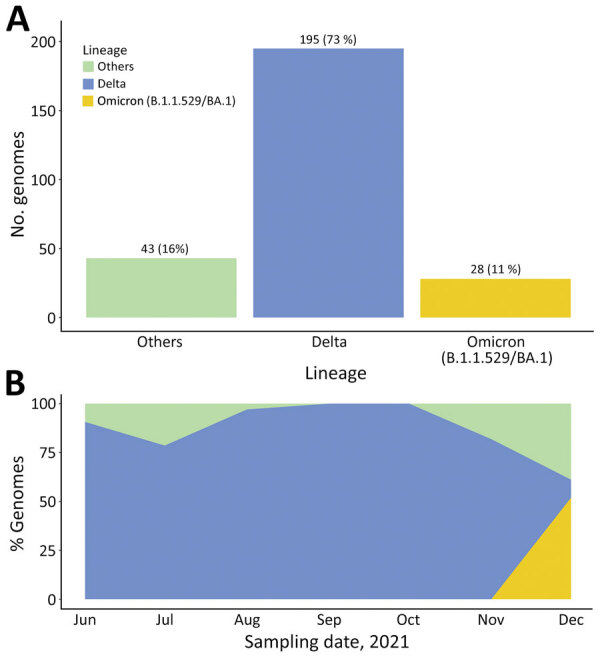
Distribution of severe acute respiratory syndrome coronavirus 2 (SARS-CoV-2) variants, Uganda, June‒December 2021. A) Distribution of SARS-CoV-2 variants from 266 samples genotyped during June‒December 2021. B) Percentage of SARS-CoV-2 variants genotyped during June‒December 2021 according to sampling dates.

Results showed that most (195, 73.3%) of the 266 SARS-CoV-2 sequences genotyped were the Delta variant (B.1.617.2 and other AY.1, AY.4, AY.33, AY.39, AY.46, AY.46.4 sublineages), a variant of concern (https://www.who.int/en/activities/tracking-SARS-CoV-2-variants; accessed January 10, 2022). Another variant of concern we identified was the Omicron variant (B.1.1.529 and BA.1 sublineage) (28, 10.5%). We also identified the Eta variant (B.1.525) (2, 0.8%) and other variants (41, 15.4%) mostly of the A and B lineages (Figure 1).

**Figure 2:**
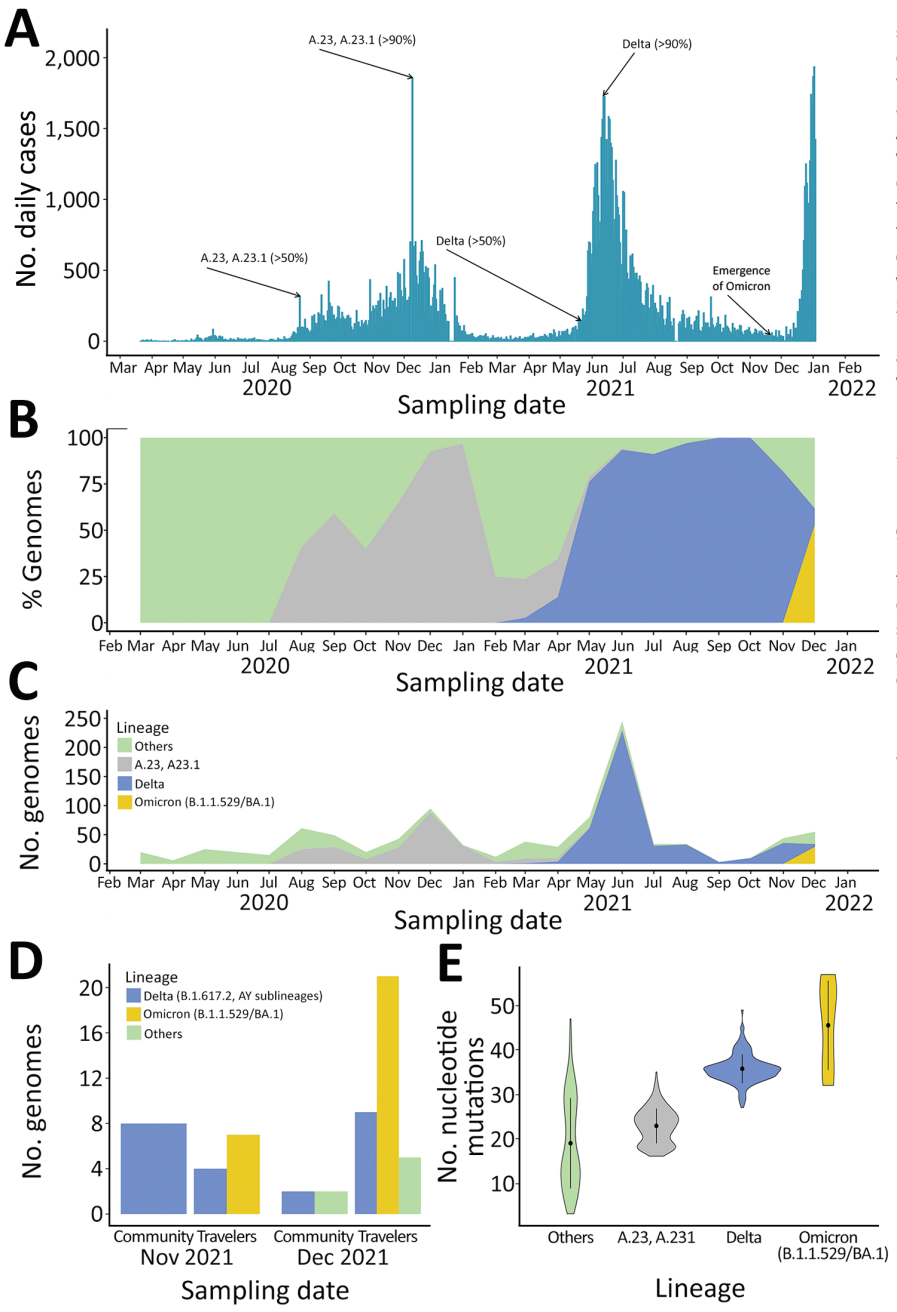
Rapid replacement of severe acute respiratory syndrome coronavirus 2 (SARS-CoV-2) variants by Delta and subsequent arrival of Omicron, Uganda, 2021. A) Coronavirus disease pandemic waves. Confirmed cases of daily coronavirus disease and trends of the SARS-CoV-2 pandemic over time. Three waves of the pandemic dominated by the A.23.1 in the first wave (December 2020‒January 2021), Delta in the second wave (May‒July 2021), and the Delta and Omicron variants in the third wave (Omicron emerged in late November 2021 and the wave began in December 2021). B, C) SARS-CoV-2 variants over time (950 genomes deposited in the GISAID database [https://www.gisaid.org] by January 10, 2022). D) SARS-CoV-2 variants during the third wave among travelers and community samples. E) Violin plots showing the distribution of whole-genome nucleotide mutations in each of the SARS-CoV-2 lineages by using the wild-type Wuhan-Hu-1/2019 isolate (GenBank accession no. MN908947) as the reference. Black dots indicate median number of nucleotide mutations. Error bars indicate interquartile ranges.

Uganda is in the third wave of the COVID-19 pandemic (Figure 2, panel A). During the first wave (December 2020‒January 2021), the A.23.1 variant dominated (*4*). During second wave (May‒July 2021) and by June 2021, Delta dominated all variants reported. We report the numbers and percentage of SARS-CoV-2 genomes generated and variants reported over time based on 950 sequences from Uganda deposited in GISAID (Figure 2, panels B, C). The first Kappa variant (B.1.617.1) was identified in March 2021. However, in June 2021, the Delta variant reached its peak and comprised >90% of all circulating variants. SARS-CoV-2 variants previously reported (*3*,*4*) have since been largely replaced by Delta, and the current third wave (began in December 2021) is dominated by Delta and the highly transmissible Omicron variant.

We performed a subanalysis of SARS-CoV-2 variants during the third wave (Figure 2, panel D). We also detected other Delta sublineages, such as AY.1 or B.1.617.2.1 (also known as Delta Plus and associated with a relatively higher transmissibility) (*6*), at a low prevalence. The AY.1 Delta sublineage has been associated with more antibody escaping properties because of the K417N mutation, which was identified in the Beta variant (*7*). We also provide the relative number of mutations for SARS-CoV-2 variants (Figure 2, panel E). We deposited all sequences generated during this study in the GISAID public database (accession nos. EPI ISL 4548461–543, EPI_ISL_6262724–47, EPI_ISL_8307285–411, EPI_ISL_8523904–5, EPI_ISL_6506618, EPI_ISL_6506627, EPI_ISL_6506639, EPI_ISL_6506648, EPI_ISL_6506655, EPI_ISL_6506666, EPI_ISL_6506674, EPI_ISL_6506689, EPI_ISL_6506697, EPI_ISL_6506706, EPI_ISL_6506713, EPI_ISL_6506721, EPI_ISL_6506726, EPI_ISL_6506738, EPI_ISL_6506747, EPI_ISL_6506751, EPI_ISL_6506760, EPI_ISL_6506767, EPI_ISL_6506773, EPI_ISL_6506784, EPI_ISL_6506791, EPI_ISL_6506802, EPI_ISL_6506812, EPI_ISL_6506824, EPI_ISL_6506829, EPI_ISL_6506835, EPI_ISL_6506841, EPI_ISL_6506844, EPI_ISL_6506851, and EPI_ISL_6506857).

## Conclusions

SARS-CoV-2 sequences deposited in GISAID from Uganda showed a rapid replacement of variants since the beginning of the COVID-19 pandemic. Genomic sequencing involving 266 samples collected during June‒December 2021 showed that the Delta variant was the dominant virus. However, the Omicron variant emerged in late November 2021 from travelers arriving through Entebbe International Airport (39.29% from South Africa, 28.57% from Nigeria, 14.29% from Kenya, 7.14% from the Democratic Republic of the Congo, 3.57% from Ethiopia, 3.57% Rwanda, and 3.57% from the United States), and Omicron community transmissions are increasing (based on PCR genotyping). Therefore, we anticipate that Delta is gradually being replaced by Omicron, which is consistent with the observed SARS-CoV-2 variants trajectory over time.

Furthermore, results from a mutation-specific SARS-CoV-2 PCR screening (*8**,**9*) suggest that Omicron, initially becoming dominant among travelers, will likely later predominate in the community. The Omicron variant has been associated with increased transmissibility and has quickly become a global concern (*10*). Speeding up genomic sequencing from prospective samples collected at points of entry and from the community will enable faster response to outbreaks as they emerge.

A major limitation of this study was suboptimal sampling. Previously, convenience sampling that targeted points of entry and outbreak hotspots was more common. Sampling prioritized mostly moderate-to-high community transmission sites and focused less on sampling low viral transmission communities. However, plans are under way to adopt effective sampling guidelines to ensure geographically representative sampling (*11**,**12*).

In summary, the SARS-CoV-2 Delta variant rapidly replaced earlier virus variants after it was introduced into Uganda. The Omicron variant has followed the same trajectory. Our results highlight the need for surveillance and infection control measures to prevent future pandemic waves. 
